# Patterns of Distress and Supportive Resource Use by Healthcare Workers During the COVID-19 Pandemic

**DOI:** 10.3390/healthcare13151785

**Published:** 2025-07-23

**Authors:** Mahiya Habib, Aaron Palachi, Melissa B. Korman, Rosalie Steinberg, Claudia Cocco, Catherine Martin-Doto, Andrea Tuka, Xingshan Cao, Mark Sinyor, Janet Ellis

**Affiliations:** 1 Sunnybrook Health Sciences Centre, Toronto, ON M4N 3M5, Canada; 2 Department of Psychology, Toronto Metropolitan University, Toronto, ON M5B 2K3, Canada; 3 Temerty Faculty of Medicine, University of Toronto, Toronto, ON M5T 2S8, Canada; 4 Toronto Police Services, Toronto, ON M5G 2J3, Canada; 5 Canadian Armed Forces, Ottawa, ON K1A 0K2, Canada

**Keywords:** COVID-19 pandemic, healthcare workers, occupational health, psychological distress, mental health resources

## Abstract

**Background/Objectives:** Healthcare workers (HCW) have increased the risk of occupational stress injuries and adverse mental health outcomes, which were exacerbated during the COVID-19 pandemic. Understanding HCW psychological distress patterns and help-seeking behaviors can inform responsive resource development that may mitigate negative outcomes in future crises. This paper provides insights on monthly trends in HCW distress and support utilization at a large Canadian hospital over a 14-month period. **Methods:** As part of a hospital-wide wellness initiative during COVID-19, the STEADY program emailed monthly confidential wellness assessments to hospital staff from April 2020 to May 2021. The assessments included screens for burnout, anxiety, depression and posttraumatic stress, types of support accessed, and demographic information. Repeated cross-sectional data were summarized as monthly proportions and examined alongside longitudinal COVID-19 data. **Results:** A total of 2498 wellness assessments were submitted (M = ~168 monthly, range: 17–945). Overall, 67% of assessments had at least one positive screen for distress. Average positive screens were 44% for anxiety, 29% for depression, 31% for posttraumatic stress, and 53% for burnout. Despite high distress, most respondents used informal supports (e.g., family/friends), highlighting limited formal support use. **Conclusions:** HCWs experienced sustained high levels of psychological distress during the COVID-19 pandemic, with burnout remaining a predominant and persistent concern. The limited use of formal support services may indicate barriers to accessing these types of supports. Our findings underscore the need for accessible and acceptable mental health supports for HCW during prolonged crises.

## 1. Introduction

Occupational stress is defined as the physical, emotional, and mental strain experienced in response to work-related pressures and demands [1]. Healthcare workers (HCW) are under immense pressure due to the potential life-or-death consequences of their work, along with overwhelming work demands [2]. The stressful nature of their jobs places HCW at a heightened risk of suffering from occupational stress injuries, including burnout, posttraumatic stress disorder (PTSD), anxiety, and depression [2,3]. These occupational stressors and reported levels of distress were heightened during and after the recent COVID-19 pandemic, resulting in elevated rates of psychological disorders, reduced staff retention, and negative outcomes, including increased medical errors, compromised patient safety, and lower quality of care due to cognitive and emotional fatigue across HCW populations [4,5]. Furthermore, high rates of burnout and trauma symptoms have been linked to reduced staff retention, compounding workforce shortages and further destabilizing healthcare systems during critical periods [6].

Burnout is defined as physical, emotional, and mental exhaustion resulting from prolonged exposure to intense stress; it is prevalent in high-demand professions such as healthcare [7]. Characterized by energy depletion, increased cynicism, mental detachment from work, and a reduced sense of professional and personal efficacy, burnout is a condition that builds over time [8]. Chronic burnout is correlated with emerging mental health challenges (i.e., anxiety, depression, and PTSD), often found among HCW populations [9].

The prevalence of PTSD in HCW is 14–15%, which is 11% higher than the general population, and it increases after large-scale health crises such as the 2003 SARS Epidemic [10]. Potentially traumatic events experienced by HCW include witnessing human suffering, sudden violent or accidental death, and risk of life-threatening illnesses and physical assault by patients [11]. The COVID-19 pandemic introduced new stressors into the healthcare environment, including fear of infection, shortage of personal protective equipment, uncertainty, short staffing and increased workload [12,13]. These stressors impacted HCW around the globe, exacerbating symptoms of PTSD, burnout, anxiety, and depression, which are associated with medical errors, decreased productivity, and compromised patient safety [13,14].

HCW in urban areas hit hardest by COVID-19 experienced alarmingly high rates of psychological distress compared to pre-pandemic levels. In one Canadian study, almost two-thirds of ICU staff experienced clinically significant psychosocial distress during the acceleration phase of the COVID-19 pandemic (6 April–30 April 2020) [14]. Similarly, in a study of frontline HCW in Ontario, nearly two-thirds of respondents reported clinically significant levels of distress during peak pandemic periods. Compared to reported rates of distress in the general Canadian population during the COVID-19 pandemic (~15% for depression and ~13% for anxiety), prevalence of distress in HCW during this time was found to be markedly higher (~19% for depression and ~18% for anxiety) [15]. Based on the overwhelming evidence for adverse mental health outcomes in HCW throughout and following the COVID-19 and the SARS pandemics, future disaster response planning should incorporate plans for targeted support during disasters and supporting HCW’s ongoing psychological well-being. Planning must take into account pre-existing systemic challenges influencing the state of hospitals and staff well-being. Prior to COVID-19, hospitals faced systemic issues which exacerbated the impact of the pandemic on hospital operations and HCW mental health [16]. Chronic understaffing and a lack of adequate mental health support have already placed HCW under significant stress pre-pandemic [17]. These conditions, coupled with increased administrative and clinical demands during the COVID-19 pandemic and ongoing resource constraints, have created an unsustainable situation of workload and occupational stress that left HCW vulnerable to burnout [18].

HCW historically underutilize organizationally offered mental health resources and other mental health supports available to them, possibly due to barriers including stigma and time constraints [19]. Determining how to best mitigate occupational stress injuries has proven challenging. Prior studies have sought to evaluate how to best address consequences of occupational stressors among HCW in response to large disasters such as COVID-19 [20,21]. Recent efforts to better support HCW mental health include expanding services such as Employee Assistance Programs (EAPs-employer-sponsored initiatives that offer employees confidential access to counseling, support services, and resources) [22], crisis lines, access to virtual counseling, workplace training programs, peer-support programming, and psychoeducational or counseling services [20,21]. An important caveat noted was that many programs implemented during the pandemic to address distress were not designed to be sustainable. The development of effective and acceptable mental health disaster response plans for future epidemics and the bolstering of overall mental healthcare for HCW require better understanding of and analysis of the longitudinal patterns of distress and mental health support use in HCW.

While much of the existing literature on HCW distress during the COVID-19 pandemic is based on either cross-sectional snapshots or qualitative insights, there remains a lack of longitudinal data capturing how patterns of distress and mental health resource use fluctuate over time, particularly those that offer insights into the experiences of HCW working in population-dense urban centers in Canada, settings that often faced the greatest COVID-19 burden and systemic strain. This knowledge gap limits our ability to design responsive and sustainable wellness interventions tailored to the evolving needs of HCW during public health crises. This study addresses these gaps by providing real-world, monthly data on HCW psychological distress and support-seeking behavior across a 14-month period in Toronto, one of Canada’s largest metropolitan cities.

The Social support, Tracking distress, Education And Discussion, communitY (STEADY) program was implemented as a quality improvement (QI) project to proactively support staff at one of Canada’s largest teaching hospitals—Sunnybrook Health Sciences Centre—during the COVID-19 pandemic. STEADY is an evidence-informed mental wellness program consisting of five key components: peer partnering, wellness assessments, psychoeducation workshops, peer-support discussions or debriefings, and community-building activities [23]. Using responses gathered from the wellness assessments, this study aimed to contribute to the literature on the negative effects of COVID-19 on HCW mental distress by outlining and contextualizing proportional trends of anxiety, depression, PTSD, burnout, and mental health resource use over time in this population of Toronto HCW during the COVID-19 pandemic.

## 2. Methods

The wellness assessment component of STEADY included screening tools for anxiety, depression, PTSD and burnout, demographic questions (i.e., age, gender, unit and position), and questions about types of mental health supports accessed (i.e., family/friends, colleagues, online). Eligibility for participation in the current study required individuals to be active hospital staff members who had completed the monthly wellness assessment. Wellness assessments were emailed monthly to all hospital staff from April 2020 until May 2021 and completed using LimeSurvey. The assessment data supported hospital staff in two ways: (1) providing staff well-being information to the organization, and (2) sending personalized wellness strategies to staff who provided their email address, outlining which screens were positive, score changes (if relevant), and suggesting potentially useful resources. This paper reports on the proportional trends that emerged from the quantitative data gathered and analyzed for reports to organizational leadership. The Sunnybrook Research Ethics Board confirmed that ethics approval was not required for this QI project.

### 2.1. Measures

*Burnout* symptoms were measured using the Single Item Burnout Scale, a validated and widely used self-survey measure [24]. Participants rated their level of burnout on a scale from 0 (no burnout) to 5 (extreme burnout). Scores of 3 or higher were considered positive.

*Anxiety* symptoms were screened using the Generalized Anxiety Disorder Scale-2 [GAD-2] [25], a widely used validated 2-item version of the GAD-7 (*r* = 0.94, *p* < 0.001) tool for symptoms of anxiety [26]. Participants rated the frequency of two DSM-5 symptoms of anxiety; (1) feeling anxious, worried, or on edge, and (2) not being able to stop or control their worry, over the past two-weeks on a scale from 0 (not at all) to 3 (nearly every day). Sum scores of 3 or higher were considered positive. Cronbach’s alpha for this study demonstrated good internal consistency (*α* = 0.87).

*Depressive* symptoms were screened using the Patient Health Questionnaire-2 (PHQ-2) [27], a validated and widely used 2-item version of the original PHQ-9 screening tool. Participants rated frequency of depressed mood and anhedonia (2 DSM-5 diagnostic criteria for depression) over the past two-weeks on a scale from 0 (not at all) to 3 (nearly every day) [27]. Sum scores of 3 or higher were considered positive for depressive symptoms. Cronbach’s alpha for this study demonstrated good internal consistency (*α* = 0.87).

*Posttraumatic Stress* symptoms were measured using the Primary Care PTSD Screen for DSM-5 (PC-PTSD-5) [28] from April to June 2020, and then the abbreviated PTSD Checklist for Civilians (PCL-C) [29] for the remaining months; as the latter provided greater specificity and detail. The PC-PTSD-5 is a 5-item validated screening tool with strong sensitivity and specificity for identifying probable PTSD in individuals in primary care settings based on DSM-5 diagnostic criteria (re-experiencing, avoidance, hyperarousal, and negative alterations in cognition and mood) [28]. Respondents answered “Yes” or “No” regarding past-month experiences of traumatic events and related symptoms, with scores of 3 or higher deemed positive [28]. Internal consistency was deemed acceptable for this study (*α* = 0.72).

The Abbreviated Posttraumatic Stress Disorder Checklist for Civilians (PCL-C) [29] is a 6-item version of the full 17-item PCL-C [30], a widely used screening tool for PTSD based on DSM-IV criteria. There has yet to be a PCL-C for the DSM-5 and this measure is still commonly used for determining presence of past-month PTSD symptoms for civilians. Each item is rated on a 5-point Likert scale ranging from 0 (not at all) to 4 (extremely). Scores of 14 or higher were considered positive for PTSD symptoms. Internal consistency within this sample was considered excellent (*α* = 0.90).

*Other*. Demographic information such as age, gender, role, and department/unit were collected. Open-ended, qualitative questions regarding staff needs were asked, and space was given for staff to share other comments (findings from the qualitative portion will be reported in a future manuscript). The remaining questions centered on what types of mental health support respondents accessed (if any), such as family and friends, colleagues, EAPs, support sessions, and Lavender alerts; alerts used in healthcare settings to identify and respond to individuals experiencing emotional distress, such as patients, families, or staff through immediate support, like counseling or chaplaincy [31].

### 2.2. Procedures

Each month from April 2020 to May 2021, staff received an email invitation containing a secure link to that month’s wellness assessment. The assessments were administered using LimeSurvey, a secure, encrypted online survey platform. Participation was entirely voluntary, and staff could complete the survey anonymously or choose to provide their email address to receive personalized feedback and wellness suggestions. Survey responses were recorded directly onto LimeSurvey and subsequently downloaded into Excel by a designated research assistant. The research assistant scored the measures, distributed individualized summaries to those who provided their email addresses, and then assigned random ID numbers to each submission to deidentify the data. This ensured that all further data handling and analysis by other research staff were conducted on anonymized datasets. The research assistant who distributed feedback was not involved in the analysis. For participants who chose to provide their email address, the same identifier was used across multiple submissions to allow tailored feedback over time; however, the dataset used for analysis remained fully de-identified, and no direct identifiers were available to those conducting the analysis.

As part of the QI process, monthly response data were aggregated and reported internally to inform organizational wellness programming. As all responses were deidentified, including repeat submissions from individuals who provided email addresses, longitudinal analyses of individual-level change were not possible. Repeated responses from the same individuals were therefore treated as independent observations, due to the deidentification procedures that limited the ability to track resubmissions across months.

To support staff, those who opted to provide their email address received individualized summaries of their screening results, which included information on any positive screens, month-to-month changes in symptoms (if applicable), and links to recommended internal or external support resources. No follow-up was required, and individuals could participate in as many or as few assessment rounds as they wished. Data were stored digitally on a hospital network with firewalls and other security and back-up measures in place. Only members on the research team had access to the data. In accordance with Sunnybrook Research Ethics Board guidelines, data will be stored for 10 years, after which, digital data (computer files) will be erased.

### 2.3. Statistical Analysis

Descriptive statistics, including means and standard deviations, were reported on four outcome variables (PTSD, anxiety, depression, and burnout) with box plots to examine proportions of cross-sectional monthly outcome patterns. Analyses were conducted using IBM SPSS Statistics version 29.0.1 [32]. Data were evaluated categorically to indicate positive or negative screens for each measure, to determine what proportion of wellness assessment submissions had positive scores for anxiety, depression, PTSD, and burnout symptoms per month. Data were grouped according to unit and position. Using frequency and percentages, types of mental health support and resources accessed by HCW were summarized to determine utilization patterns.

## 3. Results

### 3.1. Participants

A total of 2498 wellness assessments were submitted from 1 April 2020 to 30 May 2021. On average, there were 168 submissions per month (range: 17–954). In total, 6% of Sunnybrook Health Sciences Centre staff (*n* = 469) HCW provided their emails to receive personalized responses, of which 80% (*n* = 380) completed the assessment more than once. As this QI project aimed to limit the burden on busy HCW, we did not initially collect age and gender, but began to do so from June 2020, as it provided valuable information for future resource planning. Thirty-two percent (*n* = 839) and 27% (*n* = 688) of responses submitted included age and gender, respectively. Of the total submitted wellness assessments who reported their gender identity, the majority of respondents (~85%, Table 1) identified as women. Among HCW who disclosed their unit, position, and division, 61 units and 79 positions were identified. There were an average of 9.9 responses per unit (range: 2–49), and 10.4 responses per position (range: 1–300), of which the psychiatry unit provided the most responses (*n* = 94), followed by the neonatal intensive care unit (*n* = 50), and the emergency department (*n* = 49). The division with the highest proportion of positive screens overall was the Sunnybrook Research Institute, followed by St. John’s Rehab (a rehabilitation hospital at a satellite campus of Sunnybrook Health Sciences Centre), and the Odette Cancer Centre (a leading cancer treatment and research facility). Out of all positions identified, nurses (25.5%, *n* = 535), administration (7.7%, *n* = 159) and physicians (4.8%, *n* = 101) were those with the largest proportion of submissions.

### 3.2. Trends of Psychological Distress

**Figure 1:**
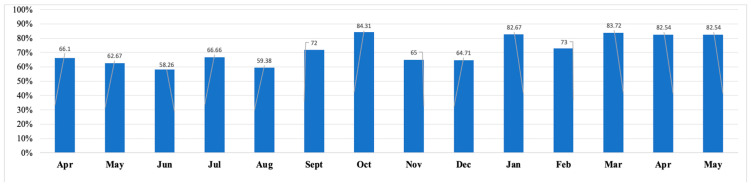
Proportion of individual submissions with any one positive screen over time.

**Figure 2:**
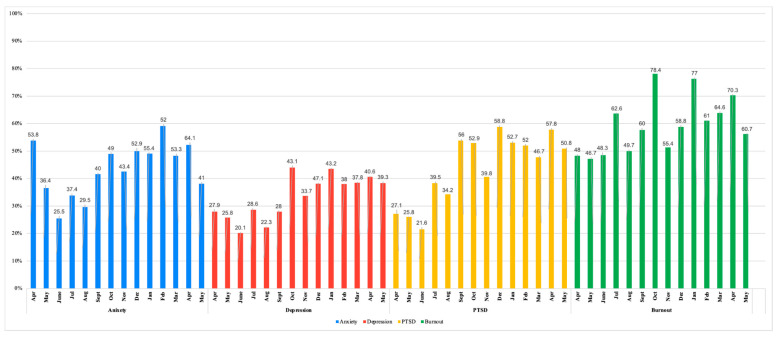
Proportion of positive screens for anxiety, depression, PTSD, and burnout over time.

Sixty-seven percent (*n* = 1673) of all submitted wellness assessments scored above the cutoff on at least one out of the four screening measures (Figure 1), with fluctuations in proportions over time (Figure 2). The proportion of submissions with at least one positive screen peaked in October 2020 (84.3%), followed by the second highest peak in January 2021 (83.7%), and then March and April 2021 (82.5%) (Table 2).

The proportions of positive screens for symptoms of anxiety and depression were fewer from May to August 2020 (by 6.90% and 3.50%, respectively). The percentage of positive screens for anxiety increased by 9% from September to October 2020 (going from 40% to 49%) and decreased by 5.60% in November 2020 (going down to 43.4%) based on submitted assessments. The proportion of positive screens for depression was greater from September to October 2020 by 15.1%, and then fewer in November by 9.4%, before seeing an increase in proportion again in December by 13.4%. The number of positive screens for PTSD from submitted assessments was fewer between May to June 2020 by 4.2% and then trended upward by 17.9% in July 2020. Similarly, the proportion of positive PTSD screens was slightly less in August 2020 by 5.3%, before spiking up by 21.8% in September 2020 and then again by 22.4% in October 2020. The presence of positive burnout screens was greater in July by 14.3%, in October 2020 by 18.4%, then again in January 2021 by 18.2%, and April 2021 by 59%. October 2020 had the highest rates of burnout, followed by January 2021 and April 2021.

The proportion of PTSD scores trended upwards from July to December 2020 and peaked in December (Table 2). GAD-2 scores demonstrated relatively consistent patterns, with the lowest scores observed in June 2020 and the highest in January 2021. PHQ-2 scores fluctuated, with the lowest scores recorded in June 2020 and the highest in January 2021. The proportion of individuals with positive depression scores was notably highest in July 2020 and March 2021. Burnout scores peaked in January 2021 and were lowest in September 2020. Throughout the study period, the proportion of individuals reporting burnout remained consistently high, reaching its highest levels in March and April 2021.

Almost half of respondents who shared their mental health resource use did not seek any support (42.4%). Of those that did, informal supports such as friends and family were the main sources accessed (Appendix A). Over 14 months of data collection, EAPs were only accessed 104 times (~4%), decreasing over the course of the study. The most common “other” supports recorded were meditation, exercise, and family physicians. Additional “other” supports accessed included mindfulness, yoga, medication, specific websites, podcasts, walks, religion, pets, and hobbies.

Recent global evidence further contextualizes our findings. An umbrella review and meta-analysis of 87 meta-analyses (encompassing over 9.4 million healthcare workers worldwide) reported high prevalence ratios for mental health challenges during the COVID-19 pandemic with depressive symptoms, anxiety symptoms, and insomnia observed in approximately 28%, 30%, and 37% of healthcare workers, respectively, and burnout affecting about 44% of this population [33]. These prevalence estimates are consistent with the elevated rates of distress, particularly burnout and anxiety, observed in our local HCW population.

## 4. Discussion

As part of the STEADY program, a total of 2498 wellness assessment submissions were obtained from staff at Sunnybrook Health Sciences Centre. Data analysis provided insights into changes in the mental well-being of a population of HCW throughout COVID-19. These results will be discussed within the context of government lockdowns and other key COVID-19 related events that occurred in Toronto parallel to data collection, to consider and understand factors that may have influenced patterns of HCW distress.

Irrespective of pandemic stage and other events during the course of the study, burnout was the most prevalent form of distress reported within this sample, with increasing proportions of positive screens trending upwards over the course of data collection, with peaks and valleys potentially related to contextual factors. This upward trend is likely due to the cumulative nature of burnout and persistent pressures of the pandemic environment.

### 4.1. First COVID-19 Government-Mandated Lockdown

The first government-mandated lockdown in Toronto in March 2020, marked a pivotal moment as little was known about the virus and public health systems were unprepared for a global health emergency of this magnitude. The Canadian government implemented stringent measures to contain the virus, including stay-at-home orders, business closures, and restrictions on non-essential activities [34], with the ultimate goal of “flattening the curve” and reducing strain on healthcare infrastructure [35].

In addition to the widespread societal disruptions and the pandemic itself, the first COVID-19 lockdown in Toronto placed an extraordinary burden on HCW. The sudden surge in critically ill patients strained hospital resources, while HCW struggled with the emotional toll of caring for patients with high mortality rates and limited treatment options [36]. HCW also faced uncertainty surrounding COVID-19 transmission and evolving treatment guidelines, possibly contributing to feelings of helplessness, fear, and psychological distress [5]. Our findings from this period highlight the profound impact of the pandemic’s early stages on HCW mental health. April 2020, the initial phase of our data collection, saw high proportions of HCW screening positive for symptoms of anxiety, burnout, and moderate levels of depression, compared to data on pre-pandemic rates for HCW [15]. Anxiety is often related to a “threat-response” compared to depression, so it is sensitive to immediate changes, especially in times of uncertainty and anticipatory dread, seen before during early pandemic periods [37]. In May 2020, while the proportion of positive screens for anxiety and burnout remained high, there was a slight decrease in depression and PTSD (by 2.1% and 1.3%, respectively) as healthcare systems were acclimating to COVID-19. This period coincided with the first COVID-19 wave peak in Toronto, where public health measures, including stay-at-home orders and the shutdown of non-essential services, were in full effect [34]. Those data align with the overwhelming distress reported in HCW during the SARS pandemic of 2003, where HCW experienced elevated levels of anxiety, depression, and PTSD in the initial stages, which were sustained throughout the course of the pandemic [38]. Uncertainty surrounding the virus, combined with shortages of personal protective equipment and concerns about infection, likely contributed to the elevated distress levels found in our study. HCW faced the dual burden of protecting themselves and their families while managing the surge of critically ill patients [5]. As seen in previous pandemic responses such as the SARS crisis, sustained exposure to stressors without adequate psychosocial support can result in long-term psychological sequelae, including heightened levels of depression, burnout, and PTSD [39]. Despite a brief reduction in COVID-19 cases by May 2020, Toronto’s hospitals remained significantly strained, and HCW continued to report high levels of burnout, similarly reported in research from the SARS epidemic [40].

### 4.2. Lifting of First Government-Mandated Lockdowns

**Figure 3:**
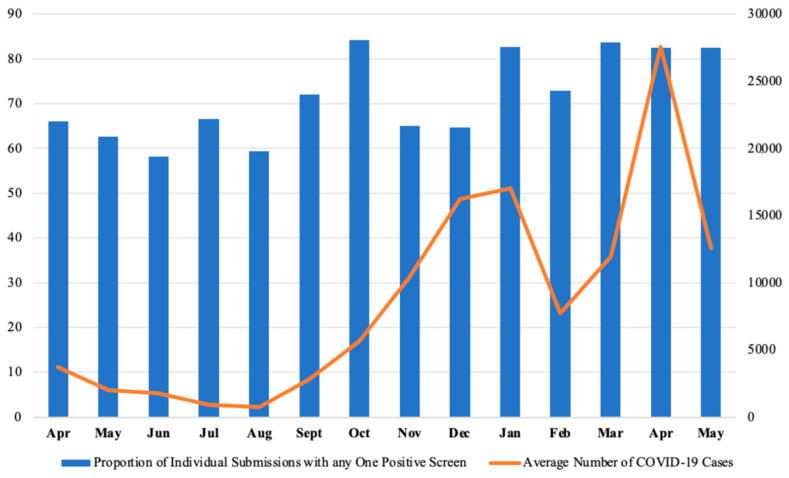
Rates of COVID-19 activity in Ontario compared to the proportion of positive screens for anxiety, depression, PTSD, and burnout over time. Note: Average numbers of monthly positive COVID-19 cases were calculated based on data openly available from the Public Health Ontario website.

The initial government-mandated lockdowns associated with the pandemic began to ease from 12 June 2020, shortly followed by the reopening of restaurants and schools at the end of June 2020 [41]. From May to June 2020, proportions of positive screens for anxiety, depression, and posttraumatic stress decreased among our HCW population, potentially relating to increased hope of returning to “normal” and excitement about the ability to spend time in-person with those outside of one’s immediate ‘bubble’ [42]. As expected, when the perceived threat [37] of the virus began to subside with falling COVID-19 cases in June 2020, so did anxiety levels within our sample. Laboratory-confirmed COVID-19 cases in Ontario (Figure 3) also declined from early June 2020, before trending upwards again from August 2020. The proportion of respondents that scored positively for PTSD, anxiety, depression, and burnout all increased from June to July 2020, potentially driven by the return of the perceived threat of COVID-19 with the lifting of government lockdowns. This anticipatory anxiety, fueled by uncertainty and fear of another wave of critically ill patients, may account for the overall increase in distress during this period. Although local lockdowns were lifted, the state of emergency (initially declared on 17 March 2020) was extended by the government of Ontario on 8 July (until 22 July) and again on 16 July (until 29 July). By August 2020, as Toronto entered Stage 3 of reopening, we observed reductions in the proportion of positive screens for anxiety, depression, PTSD and burnout, particularly anxiety, possibly aligning with reassurance from the temporary decline in case numbers and relief about the easing of restrictions.

### 4.3. Second Wave (The Beta Variant)

In September 2020, rates of COVID-19 increased again at the onset of the second wave (beta variant), with October 2020 showing the highest rates of new cases since the start of the pandemic. Unsurprisingly, October 2020 also had the largest proportion of respondents experiencing any form of distress, with 84% of respondents screening positively on at least one measure. The Ontario government postponed plans to further lift restrictions on September 8th for four weeks, although schools remained open during this time, likely contributing to increased cases [43].

Schools can serve as vectors for virus transmission, creating numerous opportunities for exposure within educational settings and the community, and introducing new transmission chains that spread into homes and hospitals [44]. On 9 October, the government announced that Toronto would go back to a modified Stage 2 for 28 days, allowing only essential activities, before another full lockdown from 23 November 2020. The decision to keep schools open may have placed HCW in a precarious position, as some may have had children attending school. This not only increased their personal risk of exposure but also added stress due to the complexities of managing childcare and fear of viral spread to family members. These conflicting responsibilities likely compounded existing strain on HCW, contributing to the rise in anxiety, burnout, and other forms of distress during this time.

The decrease in positive screens for depression in November 2020 may have been associated with anticipation of upcoming holidays [45], allowing HCW more access to their social networks-the most reported source of support accessed by this population of HCW. However, prior research suggests that holiday seasons can be more challenging for those with mental health issues, exacerbating depressive symptoms due to heightened stress, financial strain, and feelings of loneliness [45,46]. December 2020′s rise in positive depression screens may highlight this complex impact of the holidays, especially as stressors were particularly acute for HCW working long hours under intense conditions, possibly unable to fully engage in holiday traditions or spend time with family due to COVID-19 safety precautions.

Throughout December 2020, Ontario continued to break records for new daily COVID-19 infections and reached ICU capacity peaks. A provincewide shutdown was announced on 21 December, effective 26 December. The additional strain of navigating increased workload pressures alongside the backdrop of a provincewide shutdown likely compounded feelings of isolation and despair. Anxiety, PTSD, and burnout continued trending upwards after the holidays while Toronto experienced a spike in COVID-19 cases in hospitals, peaking on 3 January 2021, with 24,875 laboratory-confirmed COVID-19 cases [47].

### 4.4. Prolonged Impact of the COVID-19 Third Wave on Healthcare Worker Distress

During January–May 2021 of our study, HCW distress remained consistently high, coinciding with several events in Toronto’s pandemic timeline. In January 2021, Toronto was in strict lockdown due to a surge in COVID-19 cases, including the spread of the Beta variant, with record-high ICU admissions across Ontario. The distress among HCW during this period likely reflects the cumulative impact of prolonged exposure to high caseloads, lockdown fatigue, and of working in an overwhelmed healthcare system. Throughout these months, our data show that a substantial proportion of respondents continued to experience symptoms of anxiety, depression, PTSD, and burnout, with burnout consistently exceeding 60% during this time, peaking at 77% in January 2021 (Table 2). This suggests a persistent strain on mental health, consistent with findings in other healthcare settings facing prolonged pandemic conditions [48].

In March 2021, the Ontario Hospital Association announced that Ontario had entered the third wave of COVID-19. This third wave, which saw record-breaking numbers of new infections and hospitalizations, likely contributed to sustained high rates of psychological distress among HCW. The pressure to manage higher patient loads, while facing persistent shortages in staff and medical resources, exacerbated feelings of helplessness, anxiety, and burnout among frontline workers [13,22], relating to our findings that HCW continued to experience high levels of psychological distress, with rates of positive screens for anxiety and PTSD peaking in March (Table 2).

April 2021 saw the third provincewide shutdown driven by the Delta variant, with record-breaking numbers of new COVID-19 cases and ICU admissions. Toronto re-entered strict lockdown measures, including school closures. April 202l screening had the highest levels of PTSD reported, likely due to the unrelenting pace of patient admissions, the impact of seeing colleagues and patients affected by the new variant, and increased or prolonged fatigue after more than a year on the frontlines. Studies on prolonged disaster response show that repeated exposure to trauma and stress, especially under resource-limited conditions, is associated with increased PTSD and burnout rates among HCW [39]. In May 2021, while lockdown continued, there was a gradual decline in reported distress among HCW, coinciding with accelerated vaccine rollouts [49]. Despite this slight improvement, burnout remained high, suggesting that the prolonged strain of COVID-19 weighed heavily on HCW.

**Mental Health Support Access**

Although high rates of distress were reported in these wellness assessments, 948 of the 2498 (38%) responses indicated that no mental health resources were accessed during the study period. Throughout these stages of the pandemic, the proportion of individuals screening positively for one of the four indicators of psychological distress was consistently higher than the proportion of respondents who sought support. For instance, in April 2020, 66.1% of respondents screened positively for anxiety, depression, burnout and/or PTSD while only 38.9% accessed any form of support to address their distress. Of those who did access support, most reported the use of social support from family (52%), friends (31%), colleagues (20%), or the use of online resources (25.6%). Kisely et al. (2020) [6] report similar findings; HCW experiencing psychological distress during the COVID-19 pandemic were often reluctant to access mental health services. They identified several key barriers to utilizing formal mental health resources, including concerns about confidentiality, stigma, time constraints, accessibility, and preferences for informal coping mechanisms such as talking to family and friends. This discrepancy between those in distress and those seeking mental health supports suggests a need for targeted strategies to promote help-seeking behavior among HCW.

Over the course of the pandemic there were more responses indicating the increased use of online mental health resources, though the proportion of respondents still remained relatively low. Growing evidence indicates that hospitals might consider adjusting their EAPs and shift towards peer support or other more accessible wellness programming [50,51].

**Occupational Stress and Support Interventions**

Our findings underscore the heavy occupational stress faced by healthcare workers during the COVID-19 pandemic, aligning with broader evidence of heightened distress in the Canadian healthcare workforce [15,16]. Burnout rates in certain Canadian healthcare sectors increased to unprecedented levels (e.g., 78.7% in a survey of public health personnel) and were linked to stressors like excessive workloads, resource shortages, and lack of support [16]. These trends highlight how pandemic-related pressures, from moral distress (for example, witnessing patients die without family, providing futile care) to chronic understaffing, compounded an already challenging work environment for Canadian providers [9,11]. Consistent with this literature, the high distress observed in our study samples reflect an environment of sustained psychological strain, which poses risks not only to individual well-being but also to workforce stability if unaddressed [12,16].

Our results indicate that most healthcare providers gravitate toward informal coping avenues such as leaning on family, friends, or colleagues, rather than engaging with formal supports [21]. HCWs often report long work hours and fear of being perceived as weak or “overreacting,” which deter them from accessing available programs [20,21,48]. This hesitancy to seek formal care can become self-perpetuating, in that those struggling may delay help until distress becomes severe, at which point they may feel even less confident in the adequacy of supports or too overwhelmed to initiate use [21]. Our findings therefore align with a growing body of evidence that identifies perceived stigma, time constraints, and reliance on informal support as significant obstacles to help-seeking among healthcare providers [21,48].

Due to the limited mental health resource use, these findings carry important implications for the design of supportive interventions. Given the high levels of stress and burnout in this population, there is consensus that multi-faceted interventions are needed, ones that combine systemic changes with measures that encourage individual help-seeking [51]. Leadership and workplace culture are also critical as visible support from management, open dialogue about mental health, and policies that protect time for self-care can reduce the perceived stigma of seeking help [48,51]. Notably, lack of professional emotional support at work has been identified as a risk factor for burnout and intent to leave [15,16], therefore fostering a supportive environment is not only beneficial for well-being but may improve staff retention. Building on these findings, hospitals can take several practical steps to improve engagement with mental health support resources among staff. As HCWs already rely on trusted informal networks, supportive interventions could include peer-support programs, specialized crisis lines, and approaches that involve proactive outreach (for example, text-based check-ins) that show promise in reducing stigma and improving access for HCWs who might not otherwise seek care [21]. In parallel, formal services should also be made more accessible and acceptable. This can be achieved through embedding mental health resources directly into the workplace (such as on-site counseling or drop-in wellness sessions) and offering flexible options like confidential digital platforms that may reduce barriers to uptake [48]. Integrating support into the daily workflow through visible, low-barrier initiatives such as peer-support teams, drop-in wellness spaces, or brief wellness check-ins/huddles during the workday, can help normalize the use of support resources without requiring additional time commitments from HCW.

Tailoring interventions to specific subgroups may further improve uptake. As evident by the reduced number of men who had submitted assessments in the present study, supportive interventions may also seek to reduce help-seeking stigma for men and encourage use of support services. Given the overrepresentation of women in this sample and the underrepresentation of men, future wellness programming should consider gender-sensitive strategies that address unique barriers to help-seeking. For men, this could involve targeted outreach campaigns, mentorship by male peers, or language that aligns with values of resilience and team support rather than vulnerability [52,53,54]. Preliminary observations from our dataset also suggest that men are less likely to complete wellness assessments or report distress, consistent with broader research on gender differences in mental health help-seeking [52,53,54]. While our current data do not permit in-depth analysis due to small sample sizes, future directions include qualitative interviews and focused surveys to better understand barriers among male HCW and co-develop more inclusive support strategies. Additionally, identifying subgroups by role (e.g., nurses, physicians, administrative staff) or unit (e.g., emergency vs. rehabilitation) may allow for customization of support to their specific stressors and needs. Ultimately, our findings suggest that support strategies must be tailored to HCWs needs and routines. By combining organizational reforms (to ease undue stress) with targeted initiatives to normalize and facilitate help-seeking, future interventions can better mitigate distress in this workforce [15,51].

### 4.5. Limitations and Directions for Future Research

One of the primary strengths of this research is its real-world application as an organizational initiative aimed at supporting HCW during the COVID-19 pandemic. However, the nature of this work (as a QI project) limited this study; data collection was not designed as a repeated measures longitudinal study with a predetermined number of data collection points following the same sample over time. Instead, the assessments were offered as a supportive resource for staff, without knowing how long this resource would be offered for. The voluntary nature of the study may contribute to a self-selection or response bias, and the lack of initial demographic data collection limits the generalizability of the findings. HCWs who chose to respond may differ systematically from those who did not. Respondents might have been more distressed and seeking outlets for support, which could inflate observed distress levels. Conversely, the most overwhelmed staff might have been less likely to complete surveys due to time or burnout, leading to underestimation of true distress. In addition, the response rates varied substantially over the study period, with some months yielding very low numbers of responses. These fluctuations in monthly sample size limit the reliability of month-to-month comparisons. As a result, rigorous statistical trend analyses were not possible, and the findings should be interpreted as descriptive patterns rather than inferential conclusions. Nonetheless, the repeated cross-sectional data collected from April 2020 to May 2021 provide a comprehensive view of the evolving mental health landscape of HCW throughout different pandemic phases, highlighting what works best for the needs of HCW over prolonged crises such as COVID-19.

This project focused on one large teaching hospital in Toronto; thus, the degree of generalizability is unknown. The gender disparity in submissions, with significantly more women participating than men, reflects the gender composition of the healthcare workforce (i.e., the higher proportion of women in healthcare roles that submitted the assessments such as nursing and admin staff) [52]. As previous research suggests, men may be less likely to seek mental health support or participate in wellness assessments due to stigma, concerns about vulnerability, and societal expectations around masculinity [52,53,54,55,56,57,58]. This gender difference in help-seeking behavior can be linked to factors such as gender role socialization and mental health stigma [52,53,54,55,56,57,58]. The lower participation rates among men could mean that our data underestimate the true levels of psychological distress in HCW who are men. This may not only reflect underrepresentation in survey participation but also gendered differences in the likelihood of disclosing mental health symptoms. Research suggests that men are generally less likely to report emotional distress due to socialized norms around stoicism, self-reliance, and fear of stigma, which can lead to significant underreporting in self-assessment tools [56,57]. However, our ability to explore these differences was limited by inconsistent reporting of gender across submissions. Future studies should examine the gender disparity in help-seeking behaviors and mental health outcomes in HCW populations to help better inform the development of tailored, gender-responsive interventions that reduce barriers to access, engagement, and symptom disclosure, particularly during times of crises such as pandemics. They should also explore strategies to increase participation among HCW who are men, such as targeted outreach and reducing stigma related to mental health care, to ensure a more accurate understanding of psychological distress in this population.

Additionally, while some qualitative data were collected through open-ended survey questions about staff needs, with space provided for additional comments, these data will be analyzed and reported in a future manuscript. As a result, we were unable to integrate qualitative findings in the present study to fully contextualize the quantitative results presented here, limiting our ability to identify and interpret underlying mechanisms of observed patterns. This required us to infer context based on the broader pandemic climate. Moreover, although it is reasonable to assume that the pandemic was a primary driver of distress, we acknowledge that other external factors may have contributed to HCW symptoms. Without qualitative or longitudinal data to differentiate between pandemic-related and unrelated causes, it remains unclear to what extent the reported distress was directly attributable to COVID-19-related factors.

### 4.6. Implications

The findings from this project underscore the urgent need for acceptable and accessible mental health resources for HCW and for improving the effectiveness of ongoing wellness programming. The lack of use of formal mental health supports across samples each month may further relate to worsening distress over the study period. As family and friends were the mental health support most accessed over the course of data collection, future wellness programming could focus on social connection. Peer support may also be more acceptable than formal supports for HCW [59], considering the barrier of stigma around disclosing distress and help-seeking, and the prioritization of resilience, self-reliance, and emotional endurance within helping professions [19]. Hospitals could address this by fostering a culture that normalizes help-seeking through leadership endorsement of mental health programs, integrating mental wellness into training, and offering confidential support to reduce stigma, fear of repercussions, and enhance social connectedness. Proactive disaster response plans should address the potential long-term mental health challenges of HCW, emphasizing sustainable support and resilience-building measures.

## 5. Conclusions

This QI project provided crucial insights into the psychological distress experienced by HCW during the COVID-19 pandemic. Data from 2498 wellness assessments collected between April 2020 and May 2021 at a large teaching hospital in Toronto highlighted trends in HCW distress during COVID-19. Drawing from lessons learned from both the SARS and COVID-19 pandemics, there is clear evidence that the early stages of health crises are characterized by intense psychological distress among HCW, with lasting adverse effects on their well-being. Findings illuminated the limited use of formal mental health supports, such as EAPs, possibly due to stigma, confidentiality concerns, and time constraints. HCW who did access support relied predominantly on informal support through their social networks and online resources, underscoring the need for policies and practices that enhance the accessibility and acceptability of mental health resources for HCW. This project emphasized the need for fostering supportive environments through both formal and informal mental health resources and targeted interventions to address barriers to help-seeking. Future research should build on these findings by implementing rigorous study designs, including comprehensive demographic data collection and strategies to mitigate response bias. Additionally, further investigation into gender disparities in help-seeking behaviors and the effectiveness of various support mechanisms is essential. By understanding these trends and developing tailored strategies to address and mitigate this distress, we can better inform practices for supporting HCW and create sustainable peer-support focused wellness programs that more effectively support hospital staff in future crises.

## Figures and Tables

**Table 1:** Respondent demographic information from all submissions.

Demographic Variable	*n*	*%*
Gender		
Woman	585	84.97
Man	94	13.66
Nonbinary	3	0.44
Questioning	1	0.15
Prefer not to say	5	0.73
Age Range		
Under 19	1	0.12
20–29	153	18.23
30–39	228	27.19
40–49	195	23.25
50–59	180	21.44
60–69	47	5.60
70+	2	0.24
Responded but did not specify	33	3.93

Note: Demographic data reflect submitted responses overall rather than per respondent; thus, it is possible that the same respondents are represented more than once.

**Table 2:** Mean scores and proportion of assessments with positive scores for all screening measures, by month.

Month	Screens	*M*	*SD*	*n* Positive Screens
April 2020 (*n* = 945)	Anxiety	3.04	2.04	508
	Depression	1.79	1.85	264
	PTSD	1.59	1.57	256
	Burnout	2.61	1.00	454
May 2020 (*n* = 368)	Anxiety	2.28	1.88	134
	Depression	1.79	1.67	95
	PTSD	1.49	1.59	95
	Burnout	2.57	1.02	172
June 2020 (*n* = 329)	Anxiety	1.83	1.85	84
	Depression	1.47	1.58	66
	PTSD	1.22	1.54	71
	Burnout	2.60	1.05	159
July 2020 (*n* = 147)	Anxiety	2.23	1.81	55
	Depression	1.71	1.72	42
	PTSD	13.31	5.89	58
	Burnout	2.92	1.02	92
August 2020 (*n* = 193)	Anxiety	1.94	1.74	57
	Depression	1.58	1.66	43
	PTSD	12.31	5.91	66
	Burnout	2.63	1.14	96
September 2020 (*n* = 25)	Anxiety	2.36	1.55	10
	Depression	1.60	1.41	7
	PTSD	13.83	4.86	14
	Burnout	2.60	0.87	15
October 2020 (*n* = 51)	Anxiety	2.90	1.91	25
	Depression	2.53	1.99	22
	PTSD	14.92	5.93	27
	Burnout	3.22	0.95	40
November 2020 (*n* = 47)	Anxiety	2.63	1.85	36
	Depression	2.01	1.83	28
	PTSD	13.65	6.10	33
	Burnout	2.89	1.08	46
December 2020 (*n* = 17)	Anxiety	3.12	2.0	9
	Depression	2.59	1.58	8
	PTSD	16.18	6.78	10
	Burnout	2.94	1.25	10
January 2021 (*n* = 74)	Anxiety	3.34	2.15	41
	Depression	2.58	1.85	32
	PTSD	15.04	6.10	39
	Burnout	3.36	1.18	57
February 2021 (*n* = 100)	Anxiety	2.91	1.85	52
	Depression	2.31	1.92	38
	PTSD	14.78	6.29	52
	Burnout	3.05	1.18	61
March 2021 (*n* = 45)	Anxiety	3.12	1.80	24
	Depression	2.45	1.89	17
	PTSD	14.77	6.06	21
	Burnout	3.19	1.07	29
April 2021 (*n* = 64)	Anxiety	3.14	1.79	41
	Depression	2.63	1.83	26
	PTSD	14.89	6.12	37
	Burnout	3.14	1.11	45
May 2021 (*n* = 61)	Anxiety	2.46	1.71	25
	Depression	2.49	1.78	24
	PTSD	14.41	5.99	31
	Burnout	2.97	1.16	37

## Data Availability

The original contributions presented in this study are included in the article. Further inquiries can be directed to the corresponding author.

## Appendix A

**Table A1:** Mental health resources utilization by month.

Month	Resource	*n*	*%* of Monthly Respondents
April 2020 (*n* = 848)	Online	199	23.5
	EAP	21	2.5
	Professional	47	5.5
	One on One	-	-
	Group	-	-
	Lavender Alert	**-**	**-**
	Family/Friends	**-**	**-**
	Colleagues	-	-
	None	518	61.1
	Other	133	15.7
May 2020 (*n* = 332)	Online	71	21.4
	EAP	10	3.0
	Professional	34	10.2
	One on One	**-**	**-**
	Group	**-**	**-**
	Lavender Alert	**-**	**-**
	Family/Friends	**-**	**-**
	Colleagues	**-**	**-**
	None	191	57.5
	Other	45	13.6
June 2020 (*n* = 291)	Online	72	24.7
	EAP	6	2.1
	Professional	27	9.3
	One on One	2	0.7
	Group	10	3.4
	Lavender Alert	5	1.7
	Family/Friends	188	64.6
	Colleagues	121	41.6
	None	76	26.1
	Other	20	6.9
July 2020 (*n* = 127)	Online	28	22.1
	EAP	9	7.1
	Professional	14	11.0
	One on One	3	2.4
	Group	6	4.7
	Lavender Alert	1	0.8
	Family/Friends	76	59.8
	Colleagues	51	40.2
	None	34	26.8
	Other	5	3.9
August 2020 (*n* = 175)	Online	39	22.3
	EAP	10	5.7
	Professional	16	9.1
	One on One	4	2.3
	Group	14	8.0
	Lavender Alert	3	1.7
	Family/Friends	108	61.7
	Colleagues	58	33.1
	None	42	24.0
	Other	11	6.29
September 2020 (*n* = 22)	Online	6	27.3
	EAP	-	-
	Professional	6	27.3
	One on One	1	4.5
	Group	3	13.6
	Lavender Alert	-	-
	Family/Friends	15	68.2
	Colleagues	13	59.1
	None	5	22.7
	Other	5	22.7
October 2020 (*n* = 46)	Online	11	23.9
	EAP	5	10.9
	Professional	10	21.7
	One on One	3	6.5
	Group	2	4.4
	Lavender Alert	-	-
	Family/Friends	37	80.4
	Colleagues	22	47.8
	None	6	13.0
	Other	5	10.9
November 2020 (*n* = 76)	Online	21	27.6
	EAP	6	7.9
	Professional	18	23.7
	One on One	3	4.0
	Group	4	5.3
	Lavender Alert	-	-
	Family/Friends	54	71.1
	Colleagues	33	43.0
	None	14	18.4
	Other	12	15.8
December 2020 (*n* = 14)	Online	6	42.9
	EAP	2	14.3
	Professional	3	21.4
	One on One	-	-
	Group	2	14.3
	Lavender Alert	-	-
	Family/Friends	12	85.7
	Colleagues	7	50.0
	None	-	-
	Other	3	21.4
January 2021 (*n* = 68)	Online	18	26.5
	EAP	9	13.2
	Professional	13	19.1
	One on One	3	4.4
	Group	2	2.9
	Lavender Alert	-	-
	Family/Friends	44	64.7
	Colleagues	33	48.5
	None	15	22.1
	Other	10	14.7
February 2021 (*n* = 90)	Online	17	18.9
	EAP	10	11.1
	Professional	10	11.1
	One on One	1	1.1
	Group	7	7.8
	Lavender Alert	5	5.6
	Family/Friends	57	63.3
	Colleagues	35	38.9
	None	19	21.1
	Other	10	11.1
March 2021 (*n* = 40)	Online	14	35.0
	EAP	5	12.5
	Professional	8	20.0
	One on One	3	7.5
	Group	3	7.5
	Lavender Alert	-	-
	Family/Friends	24	60.0
	Colleagues	16	40.0
	None	7	17.5
	Other	1	2.5
April 2021 (*n* = 56)	Online	14	25.0
	EAP	6	10.7
	Professional	10	17.9
	One on One	3	5.4
	Group	30	53.6
	Lavender Alert	-	-
	Family/Friends	4	7.1
	Colleagues	16	28.6
	None	14	25.0
	Other	7	12.5
May 2021 (*n* = 53)	Online	16	30.2
	EAP	4	7.6
	Professional	12	22.6
	One on One	4	7.5
	Group	7	13.2
	Lavender Alert	-	-
	Family/Friends	34	64.2
	Colleagues	17	32.1
	None	7	13.2
	Other	5	9.4
